# Effect of Operating Conditions and Fructans Size Distribution on Tight Ultrafiltration Process for Agave Fructans Fractionation: Optimization and Modeling

**DOI:** 10.3390/membranes12060575

**Published:** 2022-05-31

**Authors:** Noe Luiz-Santos, Rogelio Prado-Ramírez, Rosa María Camacho-Ruíz, Guadalupe María Guatemala-Morales, Enrique Arriola-Guevara, Lorena Moreno-Vilet

**Affiliations:** 1 Centro de Investigación y Asistencia en Tecnología y Diseño del Estado de Jalisco A.C., Tecnología Alimentaria, Autopista Mty-Aeropuerto, Vía de la Innovación 404, Parque PIIT, Apodaca 66628, Mexico; nsantos@ciatej.mx; 2 Centro de Investigación y Asistencia en Tecnología y Diseño del Estado de Jalisco A.C., Tecnología Alimentaria and Biotecnología Industrial, Camino Arenero 1227, El Bajío, Zapopan 45019, Mexico; rprado@ciatej.mx (R.P.-R.); rcamacho@ciatej.mx (R.M.C.-R.); gguatemala@ciatej.mx (G.M.G.-M.); 3 Departamento de Ingeniería Química, CUCEI-Universidad de Guadalajara, Blvd. M. García Barragán 1421, Guadalajara 44430, Mexico; arriole@gmail.com

**Keywords:** fractionation, fine ultrafiltration, agave fructans, modeling

## Abstract

The objective of this work was to evaluate the effect of operating conditions and fructans size distribution on the tight Ultrafiltration process for agave fructans fractionation. A mathematical model of limiting mass flux transfer was used to represent the profile of concentrations over time at the outlet of a pilot scale ultrafiltration system. First, a Box-Behnken experimental design was performed for the optimization of the parameters that determine the operating conditions in their respective ranges: temperature, 30–60 °C; transmembrane pressure (TMP), 1–5 bar and feed concentration, 50–150 kg∙m^−3^, on the separation factor (*SF*) and permeate flux. Then, the validation of the model for different fructans size distribution was carried out. The results showed that for *SF*, the quadratic terms of temperature, TMP and feed concentration were the most significant factors. Statistical analysis revealed that the temperature-concentration interaction has a significant effect (*p* < 0.005) and that the optimal conditions were: 46.81 °C, 3.27 bar and 85.70 kg∙m^−3^. The optimized parameters were used to validate the hydrodynamic model; the adjustments conclude that the model, although simplified, is capable of correctly reproducing the experimental data of agave fructans fractionation by a tight ultrafiltration pilot unit. The fractionation process is favored at higher proportions of FOS:Fc in native agave fructans.

## 1. Introduction

Currently, there is a growing demand for products and functional ingredients that positively impact health, including soluble fibers and prebiotics, due to their many reported beneficial effects [1,2,3]. In this context, *Agave* plants, endemic to the American continent, contain around 13–17% (ww fresh weight) of fructans as energy reserve carbohydrates in mature plants [4]. At the industrial level, these fructans are extracted from the grinding and crushing of *Agave tequilana* Weber var. Azul heads to obtain a natural juice. This juice is filtered, deionized and decolored with a press filter, ionic exchange and active charcoal columns to finally obtain agave fructans in powder or syrup, commercially known as native fructans. Despite being a standardized process, it is essential to mention that the composition in terms of fructans size distribution or degree of polymerization (DP) can change, as they are natural polysaccharides of plant origin. It has been documented that the DP of agave fructans ranges from 3 to 42 [5] and can fluctuate according to plant species [6], region, soil, state of physical ripeness or plant age [7].

Native fructans have been studied for their beneficial effects on health, such as their prebiotic effect [8,9], effects on weight loss and body fat, their ability to decrease cholesterol and triglycerides, improve redox status [10,11] and favor calcium absorption, and their promotion of chemoprotective, immunomodulatory [12], and antioxidant effects. Their technological applications include improving sensory attributes [13] and acting as a fat replacer [14,15] and as a carrier in microencapsulation [16,17]. Some authors have shown that their properties depend on the chain size or DP. For example, concerning their prebiotic effects, the short chains, known as fructooligosaccharides (FOS), with a DP between 3–10, are more rapidly fermented by the probiotic bacteria of the microflora [18], favoring their growth in the transversal colon; this triggers a series of mechanisms involving the lipid [19] and glucose pathway [20]. On the other hand, long chains with DP ˃ 10 (Fc) are not hydrolyzed as easily by bacteria, which have been reported to produce different effects in tests with obese mice [20]. In terms of technological properties, it is also believed that larger chains are ideal for encapsulation and protection of antioxidants [16]. Therefore, obtaining fractions of agave fructans according to their DP would represent a broad market opportunity by favoring specific applications.

Several separation techniques are currently used to fractionate agave fructans at the laboratory level, based on limited acid hydrolysis [4], size exclusion [5], and precipitation with organic solvents [21]. However, these require long process times, strict control of operational parameters, many processing stages, and the use of solvents that need subsequent steps for their removal. The above means that it is expensive, difficult to scale, and unfriendly to the environment. In contrast, membrane technology is a physical process that does not use chemical agents, does not require heat, and separates the compounds of interest through a driving force.

Membrane processes are a fundamental part of industrial processes since they allow the clarification and purification of compounds of industrial interest. The pore size or nominal molecular weight cut-off (NMWCO) enables membrane processes to be classified into microfiltration, ultrafiltration (UF), nanofiltration, and reverse osmosis. The choice will depend on the type of separation required; both upstream and downstream can be recovered. In the case of fructans separation, very small solutes that fall into the range of a fine UF are considered, where tight membranes with an NMWCO of 1 to 3 kDa are required. 

Currently, polymeric membranes are more widely applied in the industry due to their low cost; however, ceramic membranes have excellent thermal and chemical stability, resisting pressure differences, compared to polymeric membranes [22,23]. Therefore, ceramic membranes can be used in processes where more aggressive cleaning processes are required; the reasons why more and more are being used in the food industry might include for protein recovery [24], the concentration of juice [25], dairy products [26], microfiltration of soy sauce [27], corn syrup clarification [28] and the sugar industry [29].

Several studies on the UF applications process have been reported to obtain FOS and inulins from various sources such as rice [30], chicory root [31], artichoke extract [32] and synthesized galactooligosaccharides [33]. For agave plants, NF studies at the laboratory level have been reported to enhance the purity of fructans by removing the low molecular weight sugars [34,35]; Flores Montaño et al. [36] and Pérez Martínez et al. [37] proposed the fractionation process by UF. In a previous study, Luiz et al. [38] showed the feasibility of obtaining fractionated fructans by Tight UF systems using a ceramic membrane on a pilot scale. However, as the processes are scaled, it is vitally important to optimize the operating conditions that allow for more efficient operations and lower costs. 

Mathematical modeling is an essential engineering tool that helps us study and understand the process phenomenon. In this case, the mass transfer models in the UF process have been suggested to predict the purification results under different operational conditions for industrial design, including FOS purity [39], yield and the decrease in permeate flux and operation time process [40]. However, scarce information exists on modeling the apparent rejection and concentration profiles of permeate and retentate streams over time, and none have been proven for the agave fructans process. The concentration profiles are considered an important challenge for agave fructans fractionation due to the observed variation in the size distribution of native agave fructans, which could affect the final ultrafiltered product.

Thus, the present work aims to optimize and evaluate the effect of temperature, TMP and feed concentration on the separation factor and flux of agave fructans fractions by a tight UF process at a pilot scale. In addition, a mass transfer mathematical model is proposed to analyze the effect of the native fructans size distribution (FOS:Fc proportions) on the apparent rejection and concentration profile, predicting the final product composition.

## 2. Materials and Methods

### 2.1. Feed Solution

For the experimental design, the feed solutions were prepared with *Agave tequilana* fructans (Olifructine^®^) kindly provided by Nutriagaves de Mexico. This mixture was a homogeneous batch of syrup with a concentration of 70 °Bx, composed of native fructans with 64.90% DP ˃ 10 (Fc), 23.77% FOS with DP between 3–10, and 11.33% mono-disaccharides (MD) DP 1–2 as glucose, fructose, and sucrose with an average DP of 16.3. Commercial native fructans from different batches and compositions were used for experimental validation. Total soluble solids concentration was adjusted using a digital refractometer (Pal-α,ATAGO, Tokyo, Japon) in °Bx, according to the experimental design level and expressed as kg∙m^−3^.

### 2.2. Membrane System and Procedures

**Figure 1:**
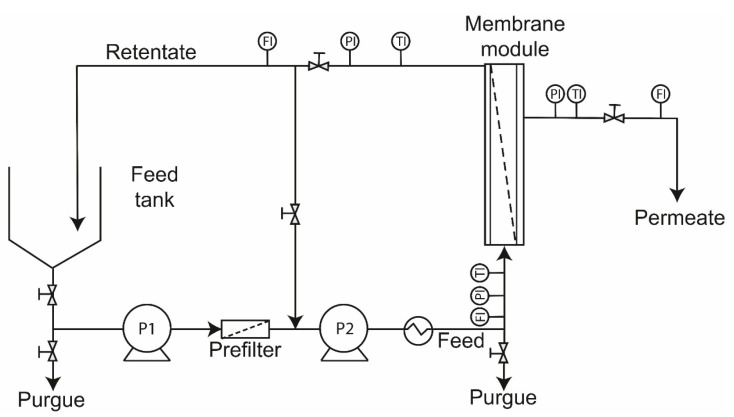
Schematic diagram of the ultrafiltration pilot unit used. P1: centrifugal pump; P2: positive displacement pump; FI: Flow Rate Indicator; PI: Pressure Indicator; TI: Temperature Indicator.

The experiments were performed in a crossflow pilot scale filtration unit (CIATEJ design) equipped with a 150 L tank. The feed flow to the membrane was driven by a positive displacement pump and a rotary piston pump connected in series (APP 1.8, Danfoss, Seoul, Korea). The system has flow, pressure and temperature sensors connected to a PLC (programmable logic controller) with a digital panel display to monitor and control the operational parameters. A heat exchanger was placed before the membrane module to reach the operating temperature. A TiO_2_ ultrafiltration membrane (inside-Céram, TAMI Industries, Nyons, France) with 1 kDa MWCO was used, tubular configuration with 39 channels with dimensions of 25 mm in diameter and 1178 mm in length, with a filtration area of 0.5 m^2^ (Figure 1). The experiments were performed in full recirculation mode; the permeate and retentate streams were returned to the feed tank. The TMP was controlled with the retentate and permeate valves, and until the system reached stable conditions (permeate flow remained constant), a sample of the permeate and feed was taken.

After each run, the membrane was cleaned with 0.4 N of NaOH at 80 °C for 30 min. After rinsing with demineralized water for 30–35 min, the membrane was washed with HNO_3_ (5 mL∙L^−1^) at 50 °C for 15 min. Subsequently, a rinse with demineralized water was performed for about 30–35 min. The permeability was measured at 1–5 bar of TMP, the tangential velocity of 3 m∙s^−1^, and a temperature of 25 °C. This permeability was compared with the initial value (35 kg∙h^−1^∙m^−2^), finding a hydraulic recovery above 95% for all the experiments.

### 2.3. Experimental Box-Behnken Design

The operating conditions: temperature, TMP, and concentration were evaluated using the Box-Behnken design, which allows for assessing several factors with a reduced number of experiments, so it is considered a robust model [41,42].

It was considered that the UF process aims to be incorporated into the current production of agave fructans. Therefore, the level of 150 kg∙m^−3^ was chosen since it is the maximum concentration obtained in industrial processes. At the same time, the temperature at which fructans are obtained is around 60 °C [43], which favors the bacterial growth restriction. Regarding the TMP used, it has been reported that low pressures favor fractionation. The previous study reported by Luiz et al. [38] showed that in the range of 1 to 5 bar of TMP, better separation is obtained, for which these values were considered in this studio. Thus, the range of operating conditions was temperature (30–60 °C), TMP (1–5 bar) and feed concentration (50–150 kg∙m^−3^). The factors, code, and variation of levels are given in Table 1.

The relationship between the factors temperature (*X*_1_), TMP (*X*_2_), feed concentration (*X*_3_), and the response variables were adjusted to linear (Equation (1)) or quadratic (Equation (2)) models using the statistical software Design Expert with an analysis of variance (ANOVA) at a confidence level of 95% (*p* < 0.005). The model with a better fit was chosen.

(1) $$\;\;Y=\beta_{0}+\beta_{1}X_{1}+\beta_{2}X_{2}+\beta_{3}X_{3}+\beta_{12}X_{1}X_{2}+\beta_{13}X_{1}X_{3}+\beta_{23}X_{2}X_{3}$$

(2) $$Y=\beta_{0}+\beta_{1}X_{1}+\beta_{2}X_{2}+\beta_{3}X_{3}+\beta_{11}X_{1}^{2}+\beta_{22}X_{2}^{2}+\beta_{33}X_{3}^{2}+\beta_{12}X_{1}X_{2}+\beta_{13}X_{1}X_{3}+\beta_{23}X_{2}X_{3}$$

where *Y* is the response variable, either separation factor or permeate flux, *β*_0_ is a constant obtained from response mean values, *β*_1_, *β*_2_, and *β*_3_ are the coefficients for the linear effect, *β*_11_, *β*_22_, and *β*_33_ are coefficients for quadratic effect and *β*_12_, *β*_13_, and *β*_23_ are the coefficients for the interaction effect.

The separation factor (*SF*) was defined by Equation (3) to evaluate the fractionation of fructans [44]

(3) $$SF=\frac{{\left(\mathrm{FOS}:F_{C}\right)}_{P}}{{\left(\mathrm{FOS}:F_{C}\right)}_{F}}$$

where the index *P* and *F* refer to the proportion of FOS and Fc in the permeate and the feed, respectively. Thus, higher *SF* values would indicate the best separation. 

The solute flux (*Ji*) through the membrane was estimated using Equation (4).

(4) $$Ji=\frac{F_{P}\cdot C_{P}}{A}$$

where *F_p_* is the mass flow in the permeate (m^3^⋅s^−1^), $C_{P\;}$is the permeate concentration (kg fructans∙m^−3^) and *A* is the membrane area (m^2^).

### 2.4. Mathematical Modeling

The effect of the native fructans size distribution (FOS:Fc proportions) on the apparent rejection and concentration profile was analyzed using a mass transfer model, solved by simultaneous equations. The polarization concentration model in terms of limiting flux is one of the most used models in ultrafiltration, which describes a concentration gradient where the molecules are deposited on the surface of the membrane, forming a layer that some authors have called gel concentration or limit concentration [45,46]. The study is based on this model under the following assumptions:

− Unsteady state.
− The transport of solutes is carried out in a single direction (flat plate).
− Osmotic pressures of macromolecular solutions are negligible
− The apparent rejection is constant throughout the process.
− The process is in limited flow conditions.

Figure 1 illustrates the ultrafiltration process of this study, for which batch-type system balance equations were used, which are widely used in concentration batch mode of ultrafiltration studies [40,47]. This system has a key consideration in the process modeling, which assumes that the solute concentration is the same throughout the system; that is, in the feed tank and feed and retentate streams. 

Where:

$V_{F}$ = Feed volume (m^3^), $F_{P}$ = is the permeate flow (m^3^⋅s^−1^),  $F_{F}$ = is the feed flow (m^3^⋅s^−1^), $F_{R}$ = is the retentate flow (m^3^⋅s^−1^) and $V_{P}$ = permeate volume (m^3^).

The change in volume of the system is due to the permeate flow collected, given by the following expression.

(5) $$\frac{dV}{dt}=-F_{P}$$

Equation (5) can be rewritten by considering the permeate flux and the membrane area.

(6) $$\frac{dV}{dt}=-J_{lim}\cdot A,\;\;\;\;\;\;\;\;\;\;\;\;\;\;\;\;\;V\;\left(0\right)=V_{0}$$

The change in concentration in the system is given by Equation (7). Rewriting Equation (7) in terms of the rejection coefficient [28] (Equation (8))

(7) $$\;\frac{dC_{i}}{dt}=-J_{lim}\cdot A\cdot \;C_{P,i}$$

(8) $$R_{o}=\left(1-\frac{C_{P,i}}{C_{i}}\;\right)$$

The material balance for solute *i* can be expressed as Equation (9). The change in concentration of solute *i* in the feed tank with respect to time is due to the solute concentration in the permeate expressed in terms of the apparent rejection coefficient.

(9) $$\frac{\;dC_{i}}{dt}=\frac{C_{i}}{V}\cdot J_{lim}\cdot A\;\cdot R_{o,i},\;C_{i}\left(0\right)=C_{i,0},\;\;\;\;\;\;\;\;\;\;\;\;\;i=\mathrm{Fc},\;\mathrm{FOS}$$

where $C_{i}$ is the concentration of solute *i,* V is the volume of retentate at any time (*t*), $J_{lim}$ is the permeate limit flux, A is the membrane area, $R_{o}$ is the apparent rejection of solute *i* (Equation (8)), $C_{P,i}$ is the permeate concentration and $C_{i}$ is the retentate concentration.

The $J_{lim}$ (Equation (10)), obtained from the model proposed by Foley et al. [45,46], is related to the polarization concentration model in terms of the rejection coefficient and limit concentration.

(10) $$J_{lim}=k\cdot ln\left(\frac{C_{lim}}{R_{o,i}\cdot C_{Fc\;\;}}-\frac{1-R_{o,i}}{R_{o,i}}\right)$$

(11) $$C_{P,i}=C_{r,i}\left(1-R_{o,i}\right)$$

where *k* is the mass transfer coefficient, $C_{lim}$ is the limit concentration at the membrane boundary layer. The value of *k* and $C_{lim}$ were obtained experimentally by plotting the natural logarithm of the concentration of the fructan mixture in a range of 50–250 kg∙m^−3^ on the abscissa axis versus the permeate flux with a TMP = 3 bar, 45 °C and a tangential speed of 3 m⋅s^−1^. The slope obtained corresponds to the mass transfer coefficient and $C_{lim}$ corresponds to the value at which the permeate flux is equal to zero.

A correlation was introduced that would allow knowing a priori the rejection coefficient as a function of the FOS:Fc ratio in the initial sample. This correlation was obtained from the characterization of 15 batches of agave fructans based on the initial FOS:Fc ratio. Subsequently, three lots were selected and ultrafiltered with a TMP = 3 bar, 45 °C and 100 kg∙m^−3^ of concentration using the FOS:Fc proportions: 0.39, 0.35 and 0.23. The results obtained from $R_{o}$ were plotted as a function of the FOS:Fc ratio.

Finally, the estimated data of the concentration versus time obtained with the model were compared with the experimental data to validate the model. Thus, the permeate concentration values of each fraction of agave fructans were calculated from Equation (11). Equations (5)–(11) were resolved simultaneously using Matlab (Matlab software, Mathworks, Inc., Natick, MA, USA). The theoretical values obtained from fructans concentration were contrasted with the experimental data obtained through the sum of quadratic errors (SSE) using Equation (12).

(12) $$\mathrm{SSE}=\overset{N}{\underset{i=1}{\sum }}{\left(Fructans\;model\;predicted_{i}-Fructans\;experimental_{i}\right)}^{2}$$

### 2.5. Determination of Size Distribution of Agave Fructans

The size distribution of fructans at each stream for experimental design was analyzed by size exclusion chromatography HPLC-SEC, using a 1220 Infinity LC System for HPLC coupled with a refractive index detector (Agilent, Alpharetta, GA, USA) and an Ultrahydrogel DP column and guard column (7.8 mm d.i. × 300 mm, Waters, Milford, MA, USA) in the stationary phase, according to the methodology proposed by Moreno-Vilet et al. [5]. This technique allows obtaining a relative abundance of Fc, FOS and MD with DP 1–2 as glucose, fructose and sucrose.

## 3. Results

### 3.1. Experimental Box-Behnken Design: Effect of Operating Conditions on the Separation Factor

A total of 16 experiments were performed according to a Box-Behnken design with four center points. Table 2 shows the experimental matrix, runs, and results of the operating conditions on the fractionation of agave fructans measured by *SF*. The *SF* presented a range between 1.75 and 2.80 and the solute flux between 0.27 and 2.6 kg∙h^−1^∙m^−2^.

In order to know which factors were statistically significant, an analysis of variance was performed. All the factors and their interactions with the evaluation factors were significant except *X*_1_ *X*_2_ and *X*_2_ *X*_3_; the interactions *X*_22_ and *X*_11_ are the most important, indicating a significant model curvature. Table 3 summarizes the sum of squares, mean square, F-value, and *p*-value. For a 95% confidence level, the *p*-value should be less than or equal to 0.05 for an effect to be statistically significant.

After eliminating statistically insignificant parameters (*X*_1_ *X*_2_ and *X*_2_ *X*_3_), the model that presented the best fit was the quadratic with R^2^ and R^2^ adjusted over 94% (See Table 3). Equation (12) represents the second-order model with actual values for *SF*.

(13) $$SF=-1.53+0.129117\cdot X_{1}+0.630625\cdot X_{2}+0.005425\cdot X_{3}+0.0001633\cdot X_{1}\cdot X_{3}-0.001533\cdot X_{1}^{2}-0.095625\cdot X_{2}^{2}-0.000077\cdot X_{3}^{2}$$

The positive values in Equation (13) mean that temperature, TMP and feed concentration: *X*_1_, *X*_2_, *X*_3_, as well as interaction *X*_1_, *X*_3_, produce an increase in *SF*, which is desirable in this process, while the negative coefficients in quadratic terms ($X_{1}^{2},\;X_{2}^{2},\;X_{3}^{2}$) produces a decrease in *SF*. 

**Figure 2:**
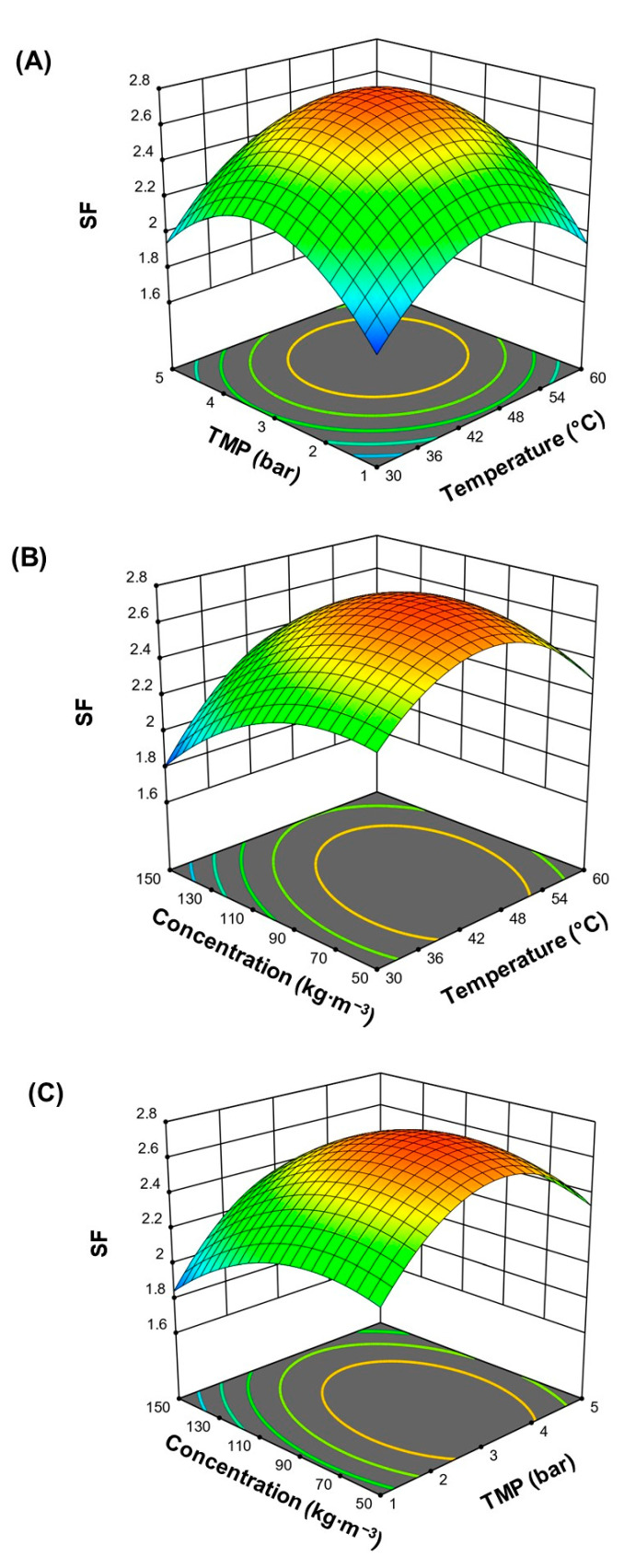
Response surface plot for *SF* as a function of (**A**) TMP, temperature and 100 kg∙m^−3^; (**B**) concentration, temperature and 3 bar of TMP; (**C**) concentration, TMP and 45 °C.

Figure 2 shows the response surface of the major effects of operating conditions studied and their interactions on *SF*. The response surface was plotted by varying two operating factors, and the third factor was kept constant at center point conditions (Level 0 in Table 1) using Equation (13). It was observed that mean values of TMP and temperature, colored red on the graph, favored the *SF* (Figure 2A,B), which agrees with results previously reported by Luiz et al. [38], where an increase in the apparent rejection coefficient for the Fc and FOS at 3 bar was observed. However, by increasing the TMP to 5 bar, the *SF* decreases. The rise in TMP produces compaction of the boundary layer on the surface of the membrane that acts as an additional barrier [48], favoring its selectivity and increasing the apparent rejection coefficient. While increasing the temperature to 60 °C, a decrease in *SF* is observed, previously reported in 2 kDa membranes [49].

Better *SF* was obtained in concentrations of 50 to 100 kg∙m^−3^, which is attributed to the extended structure (higher molar volume) of fructans adopted at these concentrations [50]; therefore, it is more difficult for the Fc fractions to cross the membrane. While increasing the concentration to 150 kg∙m^−3^, the molar volume of agave fructans decreases so the Fc can be obtained in the permeate stream. Similar results were found by Moreno-Vilet et al. [51] to evaluate the effect of concentration on the purification of inulin.

### 3.2. Effect of Operating Conditions on the Permeate Flux

The solute permeates flux (*Ji*) was used to compare the productivity as a function of temperature, TMP, and initial feed concentration. The experimental results are shown in Table 2. Table 4 shows the results of the ANOVA analysis for *Ji*, which includes the sum of squares, mean square, F-value, and *p*-value. The results showed that the best fit is obtained using a linear model, with the three factors statistically significant (*p* < 0.005) and without significant interactions.

Equation (14) shows the regression model in actual values that describe the operating conditions evaluated with linear behavior for *Ji*.

(14) $$Ji=1.16+0.3938\cdot X_{1}+0.5238\cdot X_{2}+0.3625\cdot X_{3}$$

The major productivity was shown at higher condition levels of 60 °C, 5 bar of TMP, and 150 kg∙m^−3^ of feed concentration (see Table 2). There was a positive effect of temperature on *Ji*, since it increases gradually with temperature, reaching the maximum value at 60 °C; that is, the opposite effect observed in *SF*, where it has a maximum at 45 °C but decreases at 60 °C. A common hypothesis is that increasing the temperature increases pore size, and, therefore, there is higher transit of solutes across the membrane when polymeric membranes are used [52,53]. Thus, in this case of ceramic membrane material, it is only attributed to the decrease in viscosity of the solution, which has been reported in a range of 0.048–0.051 dL∙g^−1^ for a temperature of 30 °C, which decrease as temperature increases to 60 °C [54], with a consequent rise in mass diffusion through the membrane [50,54].

There is a relationship between productivity and the initial concentration of fructans. According to the results, higher productivity could be obtained using a concentration of 150 kg∙m^−3^. This increase is attributed to a well-known phenomenon known as the concentration polarization, which is due to the accumulation of solute molecules on the membrane surface, forming a concentration gradient that facilitates the diffusion of solutes through the membrane with the aid of convective transport derived from the applied pressure [32,48].

To observe the effect of pressure, the TMP was evaluated in 1 to 5 bars. The typical behavior on the flow assessing different TMP levels can be divided into three regions [55]. The first is the linear relationship between permeate flux as pressure increases; in the second region, there is an increase in molecules on the membrane, so the linear relationship between TMP and permeate flux is no longer maintained. This inflection point is called critical flux, defined as the flux of permeate in transition between concentration by polarization and formation of the gel layer. Subsequently, the molecules form a gel on the membrane, where the increase in pressure does not cause changes in the flux, and transport in that area occurs by diffusion. In this case, the characteristic flow of the first region was observed due to a linear increase between TMP and permeate flow, so the transport in this zone is mainly by convection.

### 3.3. Optimization of Responses

In order to find the conditions that will improve the UF process, a method known as the desire function was carried out to optimize one or multiple responses. This function transforms each response level (*Y1*) into the desired score on a scale from 0 to 1. Subsequently, the individual desires are added to obtain a global desire, where the value close to 1 is considered desirable and a value close to zero undesirable.

Thus, using operational parameters within the range, maximum values of *Ji* and *SF* were searched simultaneously. Table 5 shows the optimization results considering either one or two responses. With multiple responses, an *SF* of 2.57 and *Ji* of 1.82 kg∙h^−1^∙m^−2^ of fructans were obtained with the desirability of 0.72, which, according to Svetlana et al. [56], is considered an acceptable value. The optimal values obtained for the fractionation of agave fructans are higher than those reported by Luiz et al. [38], who only used different values of TMP at 45 °C; however, similar trends were obtained.

On the other hand, considering only one response (*SF*), better results were obtained, reaching a desirability of 0.94. There is scarce literature that analyzes the separation of fractions in quantitative terms; within these, Cordova et al. [33] obtained *SF* around 1.22 to 1.94 for the separation of galactooligosaccharides and lactose, which are lower than the data obtained in this study, where values up to 2 were reached. In other studies, Rizki et al. [57] studied oligosaccharide fractionation by nanofiltration cascades with three-outlet streams to separate DP1, DP3 and DP ≥ 5 from a FOS syrup, where the *SF* between DP 3 and DP 5 reached a value of 5 when four stages are used. In this case, the obtention of two fractions: one enriched of FOS and one enriched of Fc is needed; thus, these results can assume that FOS:Fc proportion is 2.74-times enriched in permeate stream concerning feed stream, using only one membrane stage.

The quadratic model validation was carried out by analyzing four experiments on different batches of native fructans, whose results are shown in Table 6. Predicted values were calculated with Equation (13) and represent the theoretical value of *SF*. The comparison between predicted and experimental *SF* values for different operational conditions (experiments 1 and 3) supports the validity of the model to predict, including an experiment replicating at optimal conditions obtained (experiment 4).

Notably, the higher differences in *SF* values found (samples 1, 2 and 4) can be attributed to the different compositions of feed material (shown in column 2), since only experiment 3 contained almost the same composition of the experimental design runs and presented the least deviation. It demonstrated that not only are the total solid concentrations of feed solution, TMP and temperature, the factors that could affect the separation efficiency, but the composition of the feed solution is also important. Therefore, a more in-depth study is required to know how the size distribution of native fructans affects the UF fractionation process.

### 3.4. Mathematical Modeling for Different Native Fructans Size Distribution

**Figure 3:**
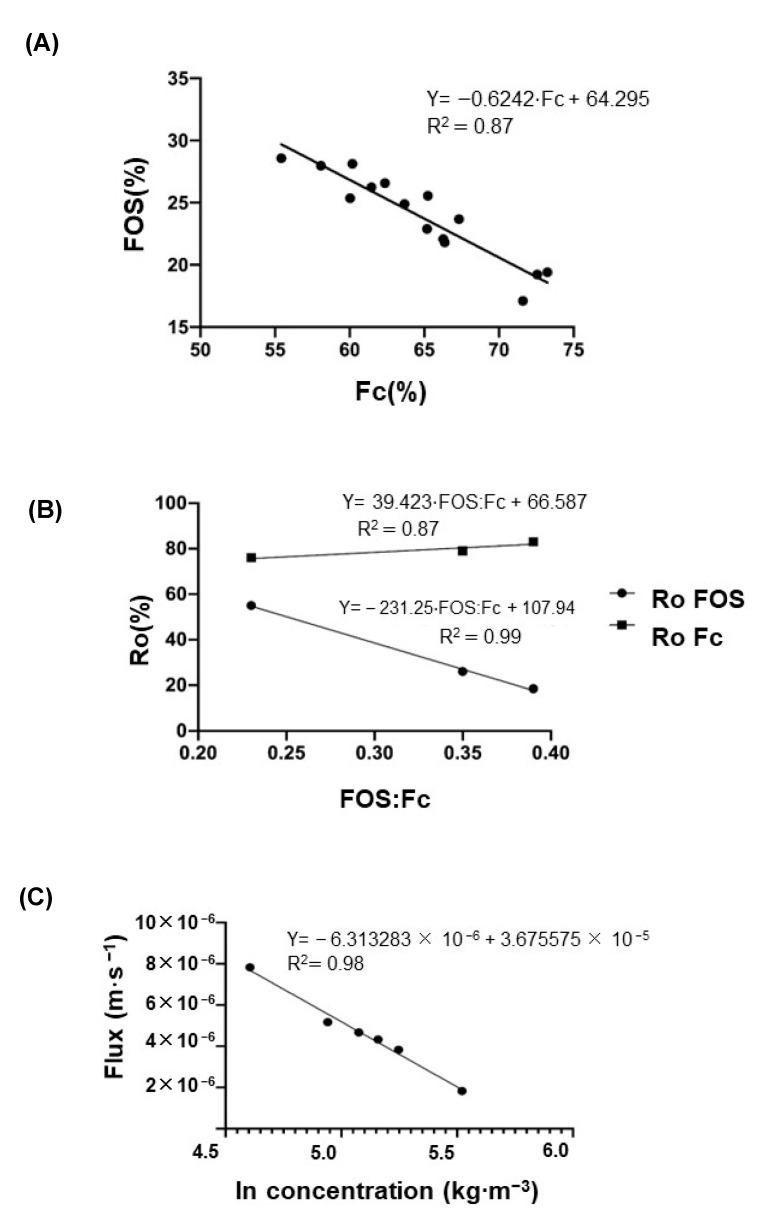
(**A**) Composition of different commercial batches of agave fructans. (**B**) Experimental rejection coefficient for different FOS:Fc ratios: 0.23, 0.35 and 0.39. (**C**) Permeate flux vs. logarithm of agave fructan concentration.

In order to analyze the effect of the native fructans size distribution (FOS:Fc proportions) on the fractionation process, 15 batches of native agave fructans were characterized, the results of which are shown in Figure 3A. The total carbohydrate content based on 100% distributed in Fc, FOS and approximately 10% MD was considered. It is observed that there is a linear relationship between the percentage of FOS and Fc obtained from 15 batches.

Figure 3B shows the results of three ultrafiltered batches that represent the FOS:Fc portions within the range found in the 15 batches. As expected, the long chains Fc present higher apparent rejection than short chains or FOS; however, this behavior is modified depending on FOS:Fc proportions. As the proportion of short chains in the feed stream increases, the *R_o_* FOS falls from 55% to 18.5%, and *R_o_* Fc remains almost constant between 76 and 83%. It means that as the size of molecules decreases, convective transport has a greater effect on its retention, and more FOS molecules pass through the membrane; thus, the fractionation process is favored at higher proportions of FOS:Fc.

To predict the behavior of the concentrations profile over time at the outlet of the pilot scale, a mathematical model of limiting mass flux transfer was used, in which it is necessary to know the parameters of Equations (5)–(11). Figure 3C illustrates the permeate flux vs. logarithm of agave fructan concentration, where the calculated value of $C_{lim}$, obtained at permeate flux equal to zero, was 337.61 kg⋅m^−3^; this value is higher than the reported for commercial inulin LGI of 198 g⋅L^−1^ [51]. These differences are attributed to the solubility, which, in the case of native inulin (2 ≤ DP ≤ 60), has reported a value of 120 g⋅L^−1^ at 25 °C [58]; after that point, a gel is formed. At the same time, this phenomenon does not occur with agave fructans [54]. On the other hand, the mass transfer coefficient *k* = 6.31⋅10^−6^ m⋅s^−1^ (Figure 3C) is close to the values found by Kuhn et al. [39] for FOS (between *k =* 9.4⋅10^−6^ and 10.6⋅10^−6^ m⋅s^−1^).

**Figure 4:**
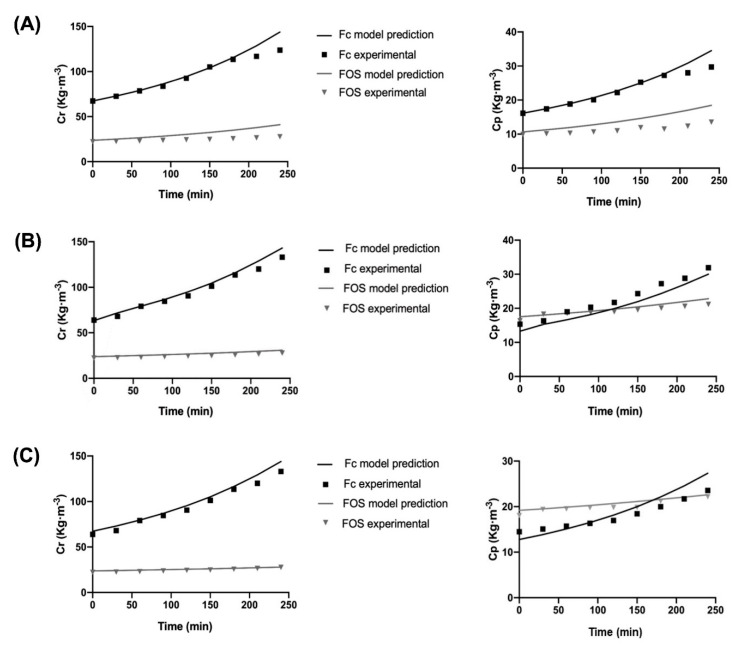
Fc and FOS concentration profile in the retentate and permeate during the UF process with an initial ratio (**A**) FOS:Fc = 0.23 (17.10%FOS:71.60% Fc), (**B**) FOS:Fc = 0.35 (23.68%FOS:67.33% Fc) and (**C**) FOS:Fc = 0.39 (24.9%FOS:63.67% Fc).

Figure 4 shows the concentration profile against the time of the FOS:Fc ratios of 0.23, 0.35 and 0.39, which were obtained experimentally and predicted. As it can be seen, there is an agreement between the experimental and theoretical data obtained from the proposed model, which validates the model for the fractionation process of agave fructans.

The results show a relationship between the initial FOS:Fc ratio and the final composition. As there is a higher proportion of FOS:Fc in the initial fructans sample, FOS concentration in the permeate is higher concerning the Fc. On the other hand, there is an increase in the concentration in the retentate stream due to the decrease in volume.

The values of SSE (Equation (12)) for Fc in the retentate streams were: 551.78, 242.32 and 296.09 for proportions 0.23, 0.35 and 0.39, respectively. It means that a greater error between the experimental and predicted data was observed with a higher concentration of Fc in the initial sample; however, similar SSE values are obtained at 0.35 and 0.39 proportions. In contrast, the values obtained of SSE for FOS in the retentate stream were: 533.55, 58.76 and 15.28 for proportions of 0.23, 0.35 and 0.39, respectively, which indicates a good fit for 0.35 and 0.39. Regarding permeate concentration profiles, for Fc, SSE values are 31.78, 39.01 and 35.6 for proportions 0.23, 0.35 and 0.39, respectively, which are very close values. While for FOS concentration, SSE values are 88.32, 8.98 and 4.91 for proportions 0.23, 0.35 and 0.39, respectively, which are very low values due to the lowest concentrations obtained at permeate of around 10 kg∙m^−3^, but clearly show a tendency for higher error as the proportion of FOS:Fc decreases or if there is a higher concentration of Fc in the initial sample.

The above deviations in some experimental data could be attributed to membrane fouling, which is not considered in the model and has been reported to reach values of around 14% of the global resistance generated in the UF process of agave fructans using ceramic systems [38]. Another hypothesis is that depending on the proportion of FOS:Fc in the initial sample, the composition also changes in the limiting layer of the membrane; therefore, different osmotic pressure values could be generated, as occurs in solutions of dextran and polyethylene glycol, which has been reported to be greater than 10–15 psi (0.68–1.03 bar) [59]. The above suggests a complex process due to low molecular weight components, so future work considering the effect of fouling in the model is needed to understand the process better.

## 4. Conclusions

It was shown that tight ultrafiltration is a viable technology for the fractionation of native agave fructans at a pilot level. The Box-Behnken design allowed us to determine the significant parameters, investigate the interaction between them, and optimize operational conditions such as temperature, TMP, and feed concentration to maximize the separation factor using a tight UF membrane of 1 kDa. The optimization of the response function allowed us to establish the conditions to obtain the maximum performance of the separation factor, which resulted in operating conditions of 46.81 °C, 3.27 bar, and 85.70 kg∙m^−3^. The results show that the fractionation process is favored at higher proportions of FOS:Fc in native agave fructans.

The limiting flux model was validated for the fractionation of agave fructans application within the studied domain to predict the concentration profiles in the permeate and retentate streams from the initial composition of the feed material. Therefore, these results show areas of opportunity for understanding mass transfer phenomena. In turn, a mathematical model is a tool that facilitates decision making in the practical operation of fructan fractionation in the industry.

## Figures and Tables

**Table 1:** Experimental range and levels of the independent variables for Box-Behnken experimental design.

Factors	Code	Variation Levels		
		−1	0	1
Temperature (°C)	*X*_1_	30	45	60
TMP (bar)	*X*_2_	1	3	5
Feed concentration (kg∙m^−3^)	*X*_3_	50	100	150

**Table 2:** Box-Behnken experimental design matrix (coded values in parentheses) and results.

Run	Temperature (°C)	TMP (bar)	Feed Concentration (kg∙m^−3^)	*SF*	*Ji* (kg∙h^−1^∙m^−2^)
	*X*_1_	*X*_2_	*X*_3_	*Y*_1_	*Y*_2_
1	45 (0)	3 (0)	100 (0)	2.80	1.350
2	30 (−1)	1 (−1)	100 (0)	1.75	0.270
3	45 (0)	3 (0)	100 (0)	2.74	1.660
4	60 (1)	3 (0)	50 (−1)	2.30	1.010
5	30 (−1)	3 (0)	150 (1)	1.80	1.130
6	60 (1)	1 (−1)	100 (0)	1.91	0.630
7	60 (1)	3 (0)	150 (1)	2.25	2.220
8	60 (1)	5 (1)	100 (0)	2.30	2.600
9	30 (−1)	5 (1)	100 (0)	1.97	1.350
10	45 (0)	5 (1)	50 (−1)	2.28	0.800
11	45 (0)	5 (1)	150 (1)	2.14	1.510
12	45 (0)	3 (0)	100 (0)	2.60	1.230
13	45 (0)	1 (−1)	150 (1)	1.90	0.790
14	45 (0)	1 (−1)	50 (−1)	2.22	0.380
15	30 (−1)	3 (0)	50 (−1)	2.34	0.560
16	45 (0)	3 (0)	100 (0)	2.70	1.110

**Table 3:** ANOVA analysis results of the Box-Behnken design for *SF*.

Source	Sum of Square	Degree of Freedom	Mean Square	F-Value	*p*-Value
*X*_1_	0.1013	1	0.1013	16.82	0.0064 *
*X*_2_	0.1035	1	0.1035	17.19	0.0060 *
*X*_3_	0.1378	1	0.1378	22.89	0.0030 *
*X*_1_ *X*_2_	0.0072	1	0.0072	1.20	0.3153
*X*_1_ *X*_3_	0.0600	1	0.0600	9.97	0.0196 *
*X*_2_ *X*_3_	0.0081	1	0.0081	1.35	0.2902
*X*_1_^2^	0.4761	1	0.4761	79.08	0.0001 *
*X*_2_^2^	0.5852	1	0.5852	97.20	0.0001 *
*X*_3_^2^	0.1482	1	0.1482	24.62	0.0025 *
Residual	0.0361	6	0.0060		
Lack of Fit	0.0149	3	0.0050	0.7040	0.6100
Pure error	0.0212	3	0.0071		
Total	1.66	15			

* Significant at *p* < 0.05, R^2^ = 97.82%, R^2^ adjusted = 94.57%.

**Table 4:** ANOVA analysis results of the Box-Behnken design for solute flux.

Source	Sum of Square	Degree of Freedom	Mean Square	F-Value	*p*-Value
*X*_1_	1.24	1	1.24	9.96	0.0083 *
*X*_2_	2.19	1	2.19	17.62	0.0012 *
*X*_3_	1.05	1	1.05	8.44	0.0132 *
Residual	1.49	12	0.1245		
Lack of Fit	1.33	9	0.1474	2.64	
Pure error	0.1675	3	0.0558		
Total	5.98	15			

* Significant at *p* < 0.05% level; R^2^ = 75.01%, R^2^ adjusted = 68.76%.

**Table 5:** Optimization results for agave fructans fractionation using a tight UF process for multiple responses and only one.

	Factors	Responses	
		*SF* and *Ji*	*SF*
Optimized coded level of variables	*X*_1_: temperatura (°C) *X*_2_: TMP (bar) *X*_3_: Concentration (kg∙m^−3^)	53.55 4.12 120.16	46.81 3.27 85.70
Predicted responses	Separation factor The flux of solute (kg fructans∙h^−1^∙m^−2^)	2.57 1.82	2.74
Overall desirability		0.72	0.94

**Table 6:** Results of validation experiments at different conditions.

Experiment	Feed Sample		Operational Conditions	Predicted *SF*	Experimental *SF*	Difference
	DP_av_	FOS:Fc				
1	15.4	0.35	45 °C, 3 bar, 100 kg∙m^−3^	2.71	3.50	0.79
2	14.6	0.39	30 °C, 5 bar, 50 kg∙m^−3^	2.05	5.78	3.73
3	16.8	0.36	60 °C, 5 bar, 50 kg∙m^−3^	1.53	1.63	0.1
4	13.5	0.44	54 °C, 4 bar, 120 kg∙m^−3^	2.56	3.25	0.69
